# Artificial cells with viscoadaptive behavior based on hydrogel-loaded giant unilamellar vesicles†

**DOI:** 10.1039/d3sc04687g

**Published:** 2023-12-04

**Authors:** Antoni Llopis-Lorente, Maaike J. G. Schotman, Heorhii V. Humeniuk, Jan C. M. van Hest, Patricia Y. W. Dankers, Loai K. E. A. Abdelmohsen

**Affiliations:** a Department of Chemical Engineering & Chemistry, Laboratory of Bio-Organic Chemistry, Eindhoven University of Technology Het Kranenveld 14 5600 MB Eindhoven The Netherlands J.C.M.v.Hest@tue.nl l.k.e.a.abdelmohsen@tue.nl; b Department of Biomedical Engineering, Laboratory of Chemical Biology, Eindhoven University of Technology Eindhoven, Het Kranenveld 14 5600 MB Eindhoven The Netherlands p.y.w.dankers@tue.nl; c Institute for Complex Molecular Systems, Eindhoven University of Technology Het Kranenveld 14, Eindhoven 5600 MB Eindhoven The Netherlands; d Instituto Interuniversitario de Investigación de Reconocimiento Molecular y Desarrollo Tecnológico, CIBER de Bioingeniería, Biomateriales y Nanomedicina, Universitat Politècnica de València, Universitat de València Camino de Vera s/n 46022 València Spain

## Abstract

Viscoadaptation is an essential process in natural cells, where supramolecular interactions between cytosolic components drive adaptation of the cellular mechanical features to regulate metabolic function. This important relationship between mechanical properties and function has until now been underexplored in artificial cell research. Here, we have created an artificial cell platform that exploits internal supramolecular interactions to display viscoadaptive behavior. As supramolecular material to mimic the cytosolic component of these artificial cells, we employed a pH-switchable hydrogelator based on poly(ethylene glycol) coupled to ureido-pyrimidinone units. The hydrogelator was membranized in its sol state in giant unilamellar lipid vesicles to include a cell-membrane mimetic component. The resulting hydrogelator-loaded giant unilamellar vesicles (designated as HL-GUVs) displayed reversible pH-switchable sol–gel behavior through multiple cycles. Furthermore, incorporation of the regulatory enzyme urease enabled us to increase the cytosolic pH upon conversion of its substrate urea. The system was able to switch between a high viscosity (at neutral pH) and a low viscosity (at basic pH) state upon addition of substrate. Finally, viscoadaptation was achieved *via* the incorporation of a second enzyme of which the activity was governed by the viscosity of the artificial cell. This work represents a new approach to install functional self-regulation in artificial cells, and opens new possibilities for the creation of complex artificial cells that mimic the structural and functional interplay found in biological systems.

## Abstract 2

Dynamic artificial cells are presented based on the synergistic combination of responsive hydrogels and a lipid membrane, resulting in regulation of the cytosolic viscosity and catalytic functions.

## Graphical abstract

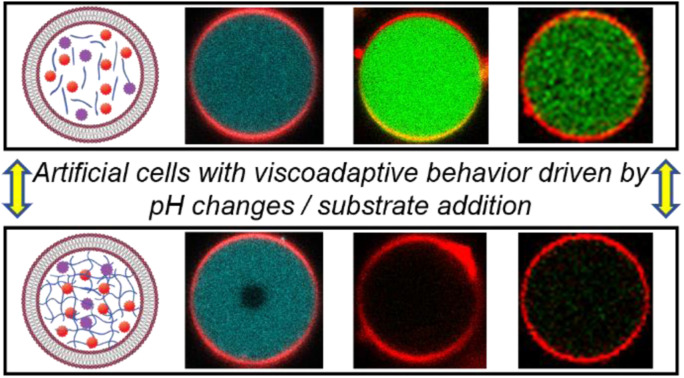

## Introduction

Artificial compartments that mimic structural and functional aspects of living cells have received increased attention from the scientific community.^1,2^ Not only do these structures allow deepening of our understanding of the functioning of biological processes in a well-defined micro-environment, they also hold potential for applications in life sciences as delivery vehicles or diagnostic tools.^3,4^ Different compartment types have been developed and studied,^5^ which accommodate processes such as protein expression,^6–8^ enzymatic reactions,^9,10^ motility^11–13^ and communication.^14–20^ A special characteristic of living systems is their ability to adapt to environmental cues, which has inspired the development of artificial cells that respond to external or internal stimuli. Adaptable structures have been mimicked, for instance, by introducing a stimulus-responsive cytoskeleton or catalytically active particles inside giant unilamellar vesicles (GUVs), that induce vesicle deformation.^21,22^ A direction which has been underexplored is the development of artificial cell systems where structural and functional changes are mutually dependent. Among the few examples that have been reported,^25–30^ two main strategies have been employed to regulate catalytic function, namely, by either utilizing responsive polymeric membranes with controllable substrate permeation;^23–26^ or by reversibly forming coacervate droplets within lipid or DNA vesicles (Table S1†).^27,28^ In this latter approach, certain reactions can be promoted in the phase-separated coacervate regions by sequestration of the reaction's components. Despite progress, the development of versatile strategies to achieve control over cytosolic processes is a major need in artificial cell research. To the best of our knowledge, there have been no examples of artificial cells with the ability to adapt their cytosolic viscosity and subsequently regulate internal processes.

In the area of adaptive supramolecular materials, the development of responsive hydrogels capable of undergoing reversible sol–gel cycles has sparked much attention in recent years due to their versatility and programmability.^29,30^ In fact, reversible control over the gelation process has been achieved in bulk materials *via* the incorporation of different enzymes that regulate the state of the gel.^31–33^ Yet, whereas hydrogels have been used as support for enzyme immobilization or as a ‘static’ chassis to construct artificial cells,^34–39^ their membranization and use as responsive functional components in artificial cells remains unexplored. A major challenge in this area is to develop a compartmentalization strategy that allows the membranization of reversible hydrogel components. Without a proper membrane, the hydrogel components will fully dissociate in the solution upon transition to the sol state without enabling sol–gel cycles. In turn, hydrogel components should not interfere with the assembly of the membrane components during the compartmentalization process.

**Fig. 1:**
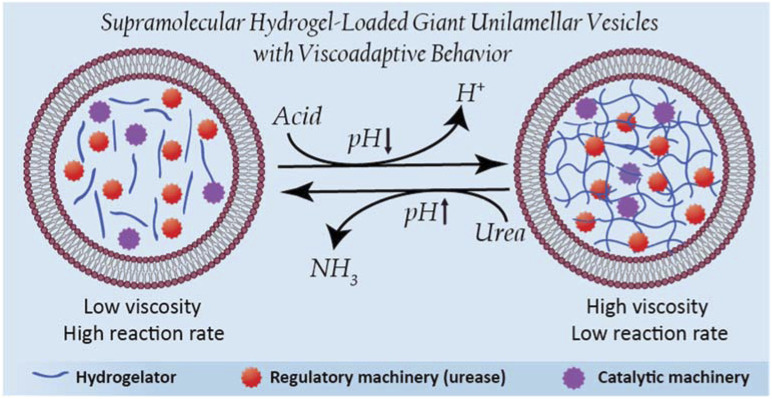
Schematic representation of hydrogel-loaded giant unilamellar vesicles as dynamic stimuli-responsive artificial cells.

In this work, we are inspired by the unique ability of natural cells to adapt their internal viscosity – a phenomenon known as viscoadaptation – in response to external and internal factors.^40^ Viscoadaptation allows cells to modulate the internal diffusion of molecules and diffusion-controlled processes in the cytosol. Reversible supramolecular interactions between biological structures (protein complexes, carbohydrates, protein–nucleic acid condensates, actin filaments) coordinate the switching between various states of viscosity. This adaptive behavior is used to regulate intracellular processes,^29^ orchestrated by internal enzyme-mediated sensing-actuation mechanisms that dictate the dynamic switching of these biological structures.^41^ Here, we present the design of viscoadaptive artificial cells based on the integration of enzymatic machinery with a supramolecular pH-responsive hydrogelator, loaded in the lumen of GUVs, yielding a new hybrid platform coined as HL-GUVs (Fig. 1). Particularly, the encapsulated hydrogel displays a low viscosity at basic pH (sol state) and a high viscosity at neutral pH (gel state).^42^ The incorporation of the regulatory enzyme urease in the HL-GUV leads, in the presence of its substrate (urea) to an increase in pH, inducing a change in viscosity. Finally, we demonstrate that viscoadaptation enables modulation of the activity of a second enzyme enclosed in the HL-GUV, thereby demonstrating the interplay between structural and functional features in a regulatory way. Altogether, we present the construction of a novel type of artificial cells based on the synergistic combination of responsive hydrogels and a lipid membrane, resulting in viscoadaptation (a mechanism so-far unexplored) to enable regulation of catalytic function.

## Results and discussion

**Fig. 2:**
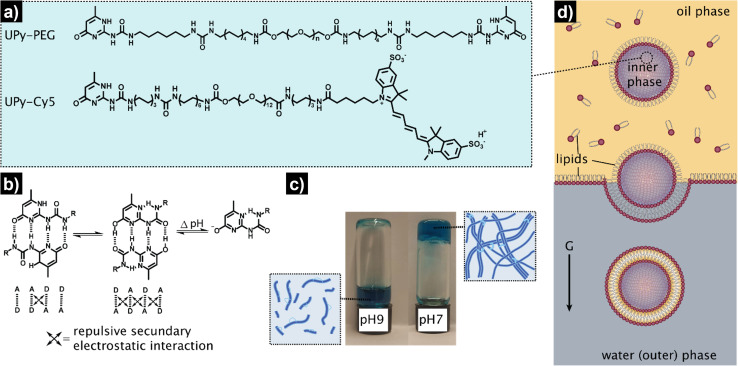
Schematic representation of the design and preparation of the HL-GUVs. (a) Chemical structures of the hydrogel building blocks: bifunctional UPy-PEG and labelled UPy-Cy5. (b) Dimerization and tautomerization mechanism of the UPy-motifs. (c) pH-responsiveness of the bulk hydrogel (UPy-PEG at 10 wt%), displaying a liquid state at basic conditions (pH 9), and a gel-state at neutral conditions (pH 7). Blue color is due to the incorporation of labelled UPy-Cy5 at 0.5 wt%. (d) Preparation of the HL-GUVs by the inverted emulsion method.

The overall procedure for the construction of our viscoadaptive stimuli-responsive artificial cell is depicted in Fig. 2. As responsive hydrogel to be included in the lumen's compartment, we prepared a switchable supramolecular hydrogelator system consisting of a poly(ethylene glycol)-based polymer coupled *via* alkyl-urea spacers to ureido-pyrimidinone (UPy) units (UPy-PEG, Fig. 2a).^43^ Multiple supramolecular interactions occur between the UPy-PEG compounds, which lead to UPy–UPy dimerization, stack and fiber formation, ultimately resulting in transient network formation. The assembly of UPy-PEG have been followed by electron microscopy in previous studies showing the formation of fibers.^42,44^ The fibers are formed through hydrogen bonding between the urea moieties of consecutive UPy-PEG compounds, being protected from the aqueous environment *via* the alkyl spacers. Crosslinking is induced by interfiber interactions through hydrogen bonding resulting in a transient network at the gelation concentration (gel state).^42,44^ As depicted in Fig. 2b, the UPy-moieties are in dynamic equilibrium between their keto and enol tautomers (both form homodimers), whereas deprotonation of the enol tautomer into the enolate under basic pH disrupts the interaction between polymeric chains and induces disassembly (sol state). The reversibility of sol–gel transitions has been previously demonstrated by rheological measurements.^42^ Fibers are found both at neutral and at basic pH, yet interfiber interactions are favored at neutral pH to induce the transition to the gel state.^42^ This therefore makes the gelation pH sensitive. The hydrogelator solution was prepared by dissolving UPy-PEG (at 10 wt%) in basic aqueous solution. This hydrogelator concentration (10 wt%) was found to be optimal in previous studies^44^ – at above this concentration (*i.e.*, 20 wt%), solubility and molecular diffusion are limited; whereas below this concentration (*i.e.*, 8% wt%), gel formation is impeded.^42,44^ In addition, we employed a small percentage (0.5%) of fluorescently-labelled hydrogelator (UPy-Cy5) to allow visualization by fluorescence confocal microscopy. Indeed, the hydrogelator solution appeared as a viscous liquid at pH 9, and rapidly formed a hydrogel when set at pH 7 (Fig. 2c). We conducted all our experiments at room temperature, yet UPy-PEG has a gel–sol transition at above 40 °C and therefore the gelled vesicles would not be not stable at elevated temperatures.^44^ It is also worth noting that UPy-PEG hydrogels are stable in physiological saline buffers such as PBS as previously demonstrated^43^ – in fact, HL-GUVs were prepared in PBS (1×) for enzyme activity experiments.

As determined in previous work, rheology of the resulting hydrogel shows viscoelastic behavior characterized with a storage modulus (G′) of 9.1 kPa (measured at 0.1 Hz).^43^ Although this is higher than the storage modulus of natural cells (0.1–0.45 kPa),^45^ the viscoelastic properties of UPy-PEG hydrogels can be tuned by introduction of collagen moieties—which offers the possibility to explore the use of other similar gelator molecules in future studies. In addition, UPy-PEG hydrogels enable the incorporation of responsive linkers in the polymer backbone, modulation of network formation by changing the length of the alkyl spacer or the molecular weight of the PEG part,^44^ and allow to access different functionalization chemistries *via* the use of monofunctional UPy additives with terminal groups such as carboxylic moieties (for coupling reactions), peptides, and markers. This chemical versatility and responsiveness together with their demonstrated biocompatibility make UPy-PEG highly appealing for the development of HL-GUVs not only for fundamental but also for biomedical applications.^43^ Notwithstanding, other types of responsive hydrogels, for instance, based on peptides could also be considered for developing HL-GUVs with different properties.

As artificial cell compartment, we chose to employ giant unilamellar vesicles (GUVs). Among several reported methods for GUV preparation, we used the droplet transfer approach (also known as inverted emulsion technique, Fig. 2d) as it typically results in high yield of GUVs with efficient encapsulation of cargo molecules using simple laboratory equipment.^46,47^ Although GUVs are usually prepared at neutral pH, we confirmed that GUVs made from a mixture of phospholipids (DOPC, POPC) and cholesterol were also formed under basic conditions (pH 9) with similar sizes (*ca.* 20 μm) and spherical morphology as compared to pH 7 conditions (Fig. S1†). In addition, we confirmed the unilamellar nature of the GUV membrane (∼4 nm) formed under basic conditions, *via* an α-hemolysin-based assay. Such basic conditions were necessary to ensure that the hydrogelator could be encapsulated in the GUVs in the sol state. To achieve the assembly of the hydrogelator-loaded GUVs (HL-GUVs), a basic hydrogelator solution (10 wt%) was added into a phospholipid mixture in paraffin oil, followed by emulsification to yield lipid-stabilized water-in-oil droplets containing the hydrogelator in the inner phase (Fig. 2d). The emulsion was then transferred on top of an aqueous phase and centrifuged to pass the droplets through a lipid-stabilized oil/water interface, yielding HL-GUVs at the bottom of the tube, which were subsequently washed and transferred to a fresh aqueous phase.

**Fig. 3:**
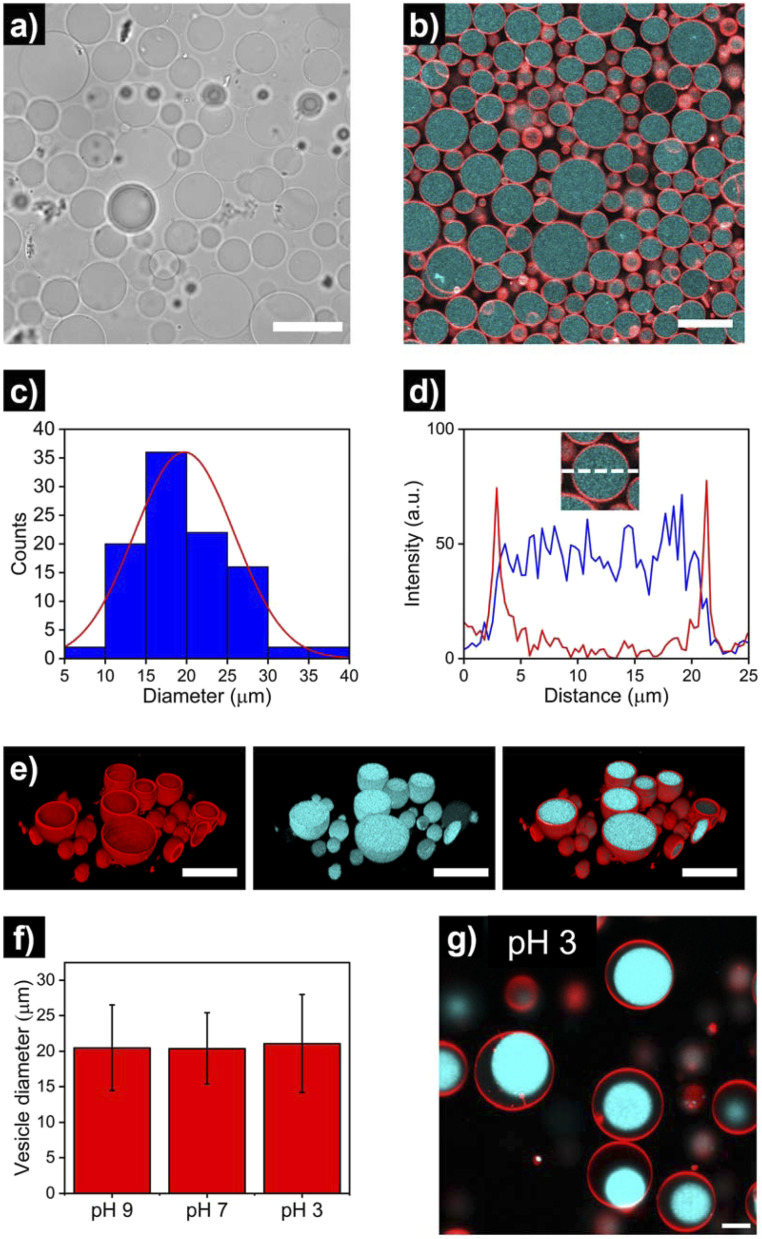
Microscopy imaging of hydrogel-loaded giant unilamellar vesicles (HL-GUVs), prepared under basic conditions (pH 9). (a) Bright field and (b) confocal micrographs (red: RhB-DOPE as marker of the lipid membrane, cyan: UPy-Cy5 as marker of the hydrogel phase) using a 63× objective. Scale bars represent 30 μm. (c) Size distribution of HL-GUVs, as determined from confocal microscopy analysis of multiple vesicles (*N* = 100). (d) Representative intensity line profile across the HL-GUV's lumen, showing the membrane's channel in red and the Cy5's channel in blue. (e) 3D confocal reconstruction showing the membrane marker, the hydrogel marker and their overlay. Scale bars represent 30 μm. (f) Average vesicle diameter of HL-GUVs under different pHs. Data expressed as mean ± s. d. (*N* = 100). (g) Confocal micrograph of HL-GUVs at pH 3 showing condensation of the hydrogel phase. Scale bar represents 5 μm.

After the assembly process, successful HL-GUV formation was confirmed using different microscopy techniques. Bright field microscopy images of HL-GUVs showed discrete spherical droplets (Fig. 3a), very similar in appearance to GUVs (Fig. S1†). Images from scanning fluorescence confocal microscopy (Fig. 3b) corroborated our observations from bright field microscopy; the phospholipid bilayer (labelled with rhodamine B-DOPE) and the hydrogelator phase (labelled with Cy5) were clearly distinguishable – confirming the successful formation of HL-GUVs. Particle size was determined by analysis of confocal images, revealing a particle distribution that mainly (>90% of particles) ranged from 10 to 30 nm, with a mean size of 20 ± 6 μm (Fig. 3c). This relatively wide size distribution is a well-known feature of the droplet transfer methodology – higher monodispersity in vesicle size could potentially be achieved using microfluidics techniques, yet this requires the use of sophisticated equipment. Moreover, intensity line profiles of the HL-GUV's lumen indicated a homogeneous distribution of the UPy-Cy5 marker (Fig. 3d). In addition, 3D imaging (reconstructed from z-stacks) of the as-prepared HL-GUVs (under basic conditions, sol state) further confirmed the integrity of the phospholipid membrane and effective encapsulation of the hydrogelator phase in the GUV lumen (Fig. 3e).

Furthermore, we looked at whether a decrease in pH of the medium (which should induce gelation at pH < 7.5) had any visible effect in vesicle morphology. As illustrated in Fig. 3f and S2 and S3,† no noticeable morphological effects (nor major changes in average size) were observed in HL-GUVs upon reducing the pH from 9 to 7. Remarkably, further reducing the pH to 3 did not affect the compartment size (Fig. 3f, S4 and S5†) but caused the appearance of hydrogel condensates inside the GUV membrane (Fig. 3g). This observation is ascribed to the formation of supramolecular crosslinks and exclusion of water from the polymer network at this low pH. In the following experiments presented below, we decided to focus on the behavior of HL-GUVs at near-neutral pH; yet it is worth noticing that this morphological behavior could potentially be tuned, leveraged and further investigated in future studies.

**Fig. 4:**
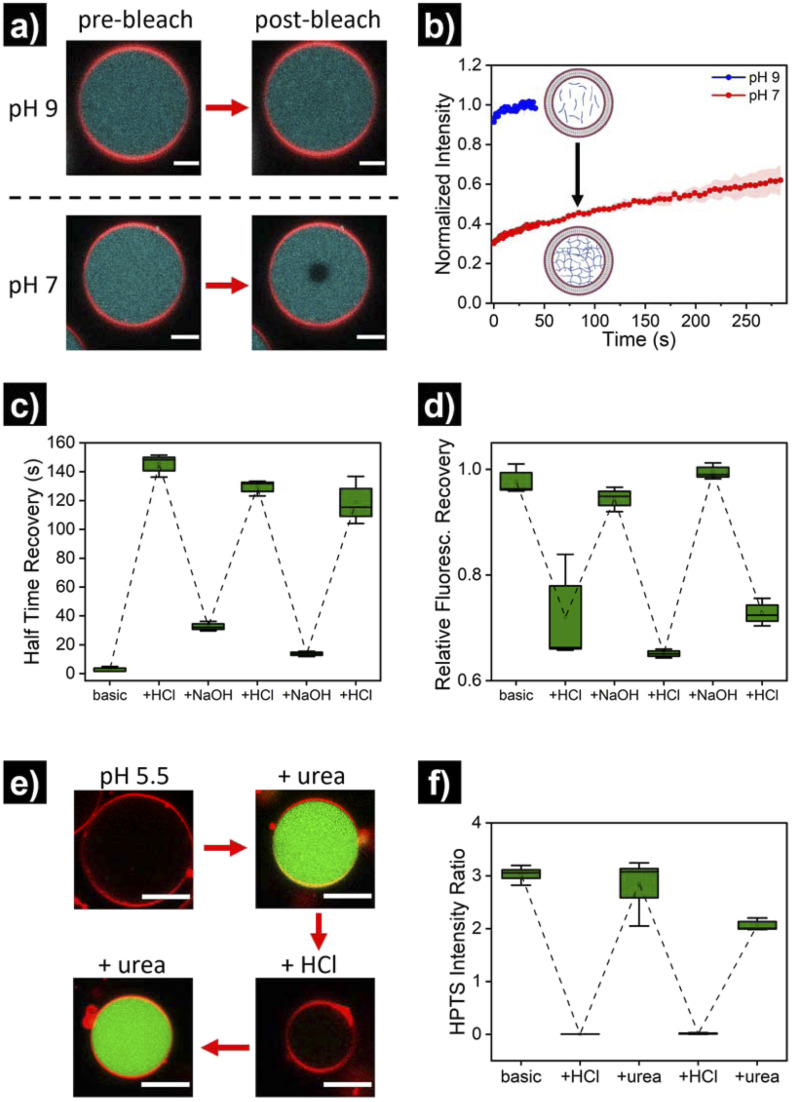
pH-switchable behavior of HL-GUVs. (a) Micrographs of FRAP experiments showing the pre-bleach and post-bleach (3 s) images at pH 9 and pH 7. Bleaching was performed by increasing the laser intensity to 100% for 10 s on the center of the vesicle. Scale bars represent 5 μm. (b) Corresponding FRAP curves at pH 9 and pH 7. Data shown as mean ± s. e. from *N* = 3. (c) Half time recovery through multiple cycles upon subsequent addition of acid and base, and (d) relative fluorescence recovery in the bleached area (10 min post-bleaching). The external pH was adjusted by adding small quantities of 0.1 M NaOH or 0.1 M HCl, and allowing 5 min for equilibration. (e) Micrographs of HL-GUVs showing HPTS fluorescence in green (excitation at 488 nm), upon subsequent addition of urea and acid to the environment. Urease (3.5 mg mL^−1^) and HPTS (0.1 mM, fluorescent pH probe) were co-encapsulated in the HL-GUV lumen. Urea was added at 25 mM final concentration and measurement were acquired 1 h after urea addition. Scale bar represents 10 μm. (f) Ratiometric (488/405) HPTS intensity ratio in the lumen of HL-GUVs, through multiple cycles upon subsequent addition of acid and urea. Data shown as box-plots from *N* ≥ 3.

Once the successful assembly of HL-GUVs was confirmed, we set out to investigate the adaptivity of the viscosity inside the lumen by triggering a sol–gel transition in response to a change of environmental pH. For these experiments, we performed fluorescence recovery after photobleaching (FRAP) measurements on the encapsulated hydrogelator. In brief, the UPy-Cy5 marker was bleached in a selected circular region (diameter of 3 μm) at the center of the HL-GUVs (by increasing the laser intensity to 100% for 10 s), followed by monitoring the fluorescence recovery (in relation to the non-bleached region). In the initial sol state, the as-prepared HL-GUVs were at basic pH (9) and photobleaching gave rise to a very fast recovery – visually, the bleached area recovered to *ca.* 100% almost immediately (in less than 1 second) (Fig. 4a). Then, we decreased the pH of the system (∼7), by addition of acid to the outer medium. At this pH, HL-GUVs exhibited a much slower recovery in the bleached area (center of the GUV), indicating slower diffusion and therefore an increase in viscosity due to the formation of supramolecular hydrogelator crosslinks (Fig. 4a and b).

In order to demonstrate reversibility of the sol–gel transition inside the GUVs, we changed the pH of the system back to its initial basic conditions and carried out multiple reversible adaptation cycles (by addition of NaOH and HCl, respectively). The corresponding half time recovery (*τ*_1/2_) values and relative fraction of fluorescent recovery were determined by single exponential fitting of the FRAP curves. The diffusion of the UPy-Cy5 marker inside HL-GUVs switched from short *τ*_1/2_ values (2–33 s) at basic pH, to long *τ*_1/2_ values (115–150 s) upon acidification through the subsequent cycles (Fig. 4c). This qualitatively resembles the behavior of natural cells upon viscoadaption – yet, reported values for *τ*_1/2_ values of macromolecules in natural cells switch from ∼1 s in the low viscosity state to ∼4 s in the high viscosity state.^40^ We believe that HL-GUVs that match more closely the behavior of natural cells could be developed in future studies, for instance, by introducing collagen moieties and employing microfluidic fabrication techniques.^43^ The relative fraction of fluorescent recovery in the bleached area was *ca.* 1 in all the three basic states (Fig. 4d), whereas for the acidified states a significant decrease in recovery was observed. These results indicate that the mobility of the fluorescent probe in the HL-GUVs lumen is significantly reduced at near-neutral conditions, whereas the mobility is restored after applying basic conditions.

Having demonstrated the structural adaptivity of HL-GUVs in response to changes in pH, we set out to investigate if this behavior could be placed under control of an internal biocatalytic regulatory mechanism. We therefore incorporated the enzyme urease, which is able to process urea and induce an upregulation of the pH. Urease (3.5 mg mL^−1^) was dissolved in the hydrogelator solution (at basic pH), prior to encapsulation in the HL-GUVs lumen. In addition, the ratiometric pH-sensitive dye 8-hydroxypyrene-1,3,6-trisulfonate (HPTS, pyranine)^48^ was co-encapsulated (as it is membrane impermeable) in order to monitor the internal pH; HPTS is fluorescent upon excitation at 405 and 488 nm, with a pH-dependency at 488 nm according to the protonation state of its hydroxyl group (pK_a_ = 7.3) (Fig. S6†). As expected, the as-prepared HL-GUVs in basic medium showed a high (488/405) intensity ratio (Fig. 4e, f and S7†) and a very fast photobleaching recovery (Fig. S8†). Then, acid was added to the external medium (pH ∼5) to induce switching to the high viscosity state, which was confirmed by the diminished HPTS intensity (*λ*_ex_ = 488 nm) and the FRAP results. At this point, urea (25 mM) was added as substrate to be converted by the enzymatic machinery to induce switching to the basic pH state. Indeed, external addition of urea led to an increase in HPTS intensity (*λ*_ex_ = 488 nm), indicating basification of the internal structure (Fig. 4e and f). Furthermore, FRAP experiments (Fig. S8†) confirmed the change inside the HL-GUVs from a lumen with high viscosity in the acidified medium, to a lower lumen viscosity upon incubation with urea. This cycle was successfully repeated by the addition of acid, followed by addition of urea. Altogether, these results confirmed that pH-regulating enzymatic machinery could be incorporated in HL-GUVs to reversibly control the structural state of the artificial cell lumen.

**Fig. 5:**
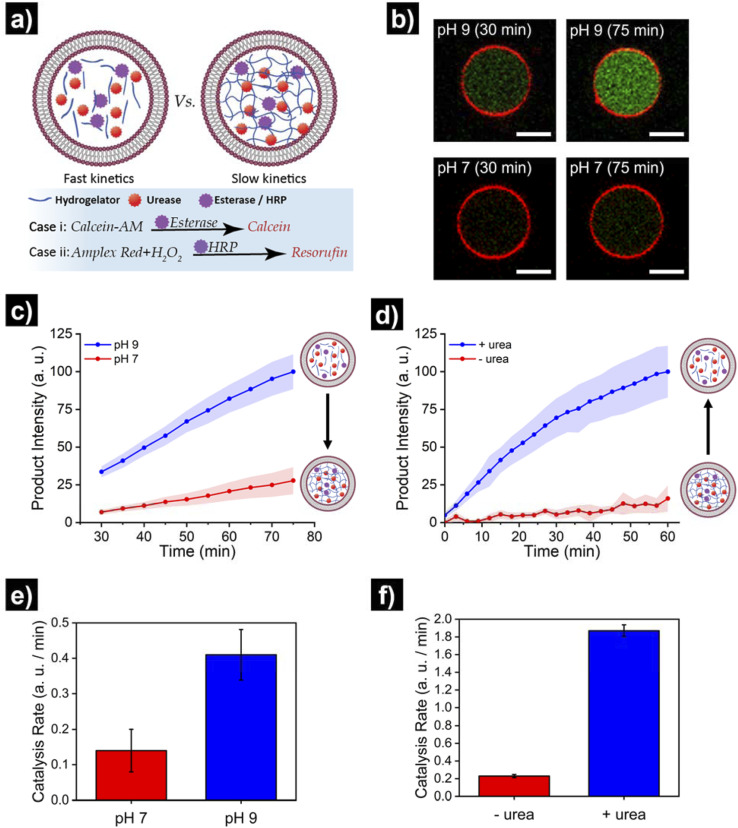
Translating structural adaptivity into functional adaptivity, where HL-GUVs act as artificial cells with viscoadaptation. (a) Schematic depicting the viscoadaptation process. (b) Micrographs showing calcein-AM conversion (into green-fluorescent calcein) in HL-GUVs (containing esterase) at pH 7 and pH 9 in PBS 1×. (c) Kinetics of product conversion by HL-GUVs containing esterase (as catalyst that converts calcein-AM into fluorescent calcein) at pH 7 and pH 9 in PBS 1×. Calcein-AM added at 400 nM. Calcein was excited at 488 nm and emission was recorded at 500–535 nm. Data is represented as mean ± s. e., *N* ≥ 8. (d) Kinetics of product conversion by HL-GUVs containing urease (as internal hydrogel-regulatory machinery) and peroxidase (HRP, as catalyst that converts Amplex Red into fluorescent resorufin) set at pH 7 in PBS 1× in the absence and presence of urea (25 mM final concentration). Amplex Red was added at 10 μm and conversion monitored using a plate reader. Data is represented as mean ± s. e., *N* = 3. (e) Catalysis rates for the esterase/calcein-AM reaction in HL-GUVs in PBS (1×) at pH 7 and pH 9. (f) Catalysis rates for the HRP/Amplex Red reaction in HL-GUVs in PBS (1×) at pH 7 in the absence of urea, and after addition of urea (25 mM). Catalysis rates were obtained from the slope of the time-dependent measurements.

Finally, we set out to showcase the translation of control over viscoelastic features of HL-GUVs into functional adaptivity by regulating catalytic processes inside the lumen (Fig. 5a). To demonstrate this concept, we prepared HL-GUVs loaded with esterase as catalyst and monitored the conversion of non-fluorescent calcein-AM (membrane permeable substrate) into green-fluorescent calcein (membrane impermeable) (as product of esterase-mediated hydrolysis).^49^ To evaluate the effect of the hydrogelator state on product formation, experiments were performed in phosphate buffer at pH 7 (gel state) and at pH 9 (sol state) and calcein intensity was monitored by confocal microscopy (Fig. 5b). HL-GUVs showed a clear difference in calcein formation over time according to the gel state, with a much slower conversion at neutral pH in comparison to pH 9 (Fig. 5c). In contrast, esterase-loaded GUVs (without hydrogel) showed a very similar increase in calcein intensity at pH 7 and at pH 9 (Fig. S9†), demonstrating the key role played by the hydrogelator in regulating metabolic activity. Then, we tested if urease-mediated hydrogel regulation could control the activity of the second enzyme. As the read-out of the esterase/calcein-AM reaction was compromised (due to calcein-AM degradation by ammonia), we switched to the peroxidase/Amplex Red reaction. We prepared HL-GUVs containing urease (as internal hydrogel-regulatory machinery) and peroxidase (as catalyst) (Fig. 5d). We set these HL-GUVs at pH 7 and then monitored the conversion of Amplex Red in the absence and presence of urea (as molecular signal to induce switching to the sol state). As a control, we first performed an experiment in GUVs without hydrogelator at different pHs. In this control experiment, both at pH 7 and 9 at HRP was able to effectively convert its substrate (with higher activity at pH 7, Fig. S10†). In contrast, product formation was slowed down in HL-GUVs at pH 7 due to the gel state, whereas a notably higher product intensity was registered in the presence of urea (Fig. 5d). The change in viscoelastic properties of the HL-GUVs lumen induced a 2.9-fold increase in the catalysis rate for the esterase/calcein-AM reaction from pH 7 to pH 9 (Fig. 5e), whereas the urease-driven process induced an 8.1-fold increase in the catalysis rate for the peroxidase/Amplex Red reaction upon supply of urea (Fig. 5f). Altogether, these results demonstrate that HL-GUVs are able to regulate internal catalytic processes according to the viscosity of the cytomimetic environment, which by itself is placed under enzyme-regulated control. This regulation of enzymatic activity imbued by changes in the viscosity of the HL-GUV lumen could have two major contributions: diffusion-based inhibition and chemical inhibition (considered as a change in the properties of the enzyme's environment). The mechanistic investigation of such phenomena is an interesting prospect for future studies.

## Conclusions

In summary, we present the compartmentalization of pH-switchable supramolecular hydrogels in lipid vesicles as an approach to construct artificial cells with viscoadaptive behavior. We loaded a hydrogelator based on UPy moieties into giant unilamellar vesicles (HL-GUVs) at high pH (to have the hydrogelator in its sol state). The (supramolecular) structural responsive properties of the hydrogelator in the HL-GUVs lumen resulted in an increased viscosity at neutral pH (where the hydrogelator was in its gel state) and low viscosity at high pH. The HL-GUVs could reversibly switch between low and high viscosity state when the pH of the environment changed. The variation in viscosity was placed under enzymatic control by the encapsulation of the pH-regulatory enzyme urease. With this system we were able to show viscoadaptation by including a second enzyme of which the activity was regulated by the variable viscosity of the GUV lumen. Overall, our work demonstrates the possibility to construct synthetic microsystems that integrate dynamic structural and functional adaptation, which provides us with a novel platform to fabricate responsive viscoadaptive artificial cells with internal regulatory mechanisms.

## Data availability

Experimental details and additional data are available in the ESI.† Any other data supporting this article are available from the corresponding authors upon request.

## Author contributions

A. L.-L. and M. J. G. S. performed the experiments and analysed the data. H. V. H. optimised and performed the peroxidase experiments. A. L.-L., M. J. G. S., and L. K. E. A. wrote the manuscript. All authors revised and provided feedback on the manuscript. The manuscript was written through contributions from all authors. All authors agreed to its publication.

## Conflicts of interest

There are no conflicts to declare.

## Supplementary Material

SC-015-D3SC04687G-s001
