# Supplementary material for: Artificial cells with viscoadaptive behavior based on hydrogel-loaded giant unilamellar vesicles
Source: Chem Sci. 2023 Dec 4;15(2):629–38. doi: 10.1039/d3sc04687g (PMC10763548; doi:10.1039/d3sc04687g)
Supplement: SC-015-D3SC04687G-s001 [file SC-015-D3SC04687G-s001.pdf]

Electronic Supplementary Material (ESI) for Chemical Science.
This journal is © The Royal Society of Chemistry 2023

Supporting Information for

Artificial Cells with Viscoadaptive Behavior Based on Hydrogel-Loaded Giant Unilamellar Vesicles

Antoni Llopis-Lorente,[†a,c,d] *Maaike J. G. Schotman,*[†b,c] *Heorhii V. Humeniuk,*[a,c] *Jan C. M. van Hest,*[a,b,c]* *Patricia Y. W. Dankers,*[b,c]* *and Loai K. E. Abdelmohsen* [a,c]*

[a] Departments of Chemical Engineering & Chemistry, Laboratory of Bio-Organic Chemistry, Eindhoven University of Technology, Het Kranenveld 14, 5600 MB Eindhoven, The Netherlands.

[b] Department of Biomedical Engineering, Laboratory of Chemical Biology, Eindhoven University of Technology, Eindhoven, Het Kranenveld 14, 5600 MB Eindhoven, The Netherlands.

[c] Institute for Complex Molecular Systems, Eindhoven University of Technology, Het Kranenveld 14, 5600 MB Eindhoven, Eindhoven, The Netherlands.

[d] Instituto Interuniversitario de Investigación de Reconocimiento Molecular y Desarrollo Tecnológico, Universitat Politècnica de València, Universitat de València, Camino de Vera s/n, 46022 València, Spain; CIBER de Bioingeniería, Biomateriales y Nanomedicina.

Email: J.C.M.v.Hest@tue.nl; P.Y.W.Dankers@tue.nl; L.K.E.A.Abdelmohsen@tue.nl.

Materials

Phospholipids (1,2-dioleoyl-sn-glycero-3-phosphocholine (DOPC), 1-palmitoyl-2-oleoyl-sn-glycero-3-phosphocholine (POPC), 1,2-dioleoyl-sn-glycero-3-phosphoethanolamine-N-(lissamine rhodamine B sulfonyl) (DOPE-RhB), 1,2-distearoyl-sn-glycero-3-phosphoethanolamine-N-[biotinylpoly(ethylene glycol)- 2000] (DSPE-PEG)) were obtained from Avanti Polar Lipids. Paraffin oil (0.86 g/cm^3 at 20 °C) was obtained from JT Baker. Cholesterol, α-hemolysin, urease (from jack bean, type III), 8-hydroxypyrene-1,3,6-trisulfoate (HPTS, pyranine), esterase (from porcine liver), peroxidase (from horseradish, HRP), calcein-AM, Amplex Red and H_2O_2 were obtained from Sigma-Aldrich. The hydrogelator UPy-PEG with M_n PEG = 10 kg mol^{-1} was synthesized by SyMO-Chem BV (Eindhoven, the Netherlands), as previously described.[1] The monofunctional UPy-Cy5 was synthesized as described previously.[2] Ultrapure water (Milli-Q) was purified on an EMD Millipore Milli-Q integral Water Purification system. 1x and 5x phosphate buffered saline (PBS) was prepared using PBS tablets (Sigma Aldrich), which was filtered before use (MF-Millipore Membrane filter, 0.45 μm pore size). MQuant® pH-indicator paper was obtained from Merck.

Instrumentation

Confocal fluorescence experiments were performed using a Leica TCS SP8 inverted confocal microscope (Leica microsystems) equipped with a 63x objective (water or glycerin immersion objective). For DOPE-RhB imaging (membrane marker), a 552 nm laser and HyD detector (565-610 nm) were employed. For Cy5 imaging (hydrogelator marker), a 638 nm laser and PMT detector (650-715 nm) were employed. 18-well glass-bottom chamber slides (ibidi GmbH) were used for vesicle visualization, which were treated with 1 mg/mL BSA in Milli-Q for >30 min, followed by washing with Milli-Q. Plate reader experiments were performed using a Tecan MC-Spark plate reader and 96 flat transparent Nunc plates. A potentiometric pH meter (FiveEasy Plus FEP20) equipped with a micro electrode (Mettler Toledo) was employed to set the pH of the working solutions. Micrograph images were quantified and processed with Fiji, a program developed by the NIH and available as public domain software at https://imagej.net/Fiji.

Hydrogel precursor preparation

The hydrogel precursor was prepared by dissolving UPy-PEG (20 mM, 20 wt%) in basic Milli-Q (pH ~11 adjusted with 1M NaOH) at 70 °C for 1h. After dissolving, the pH was

adjusted to *ca.* 10 with 1M HCl or 1M NaOH. For visualization purposes, monofunctional UPy-Cy5 was added from a stock (5 mg/mL in DMSO) to the dissolved hydrogelator at a concentration of 100 μM. Hydrogelator solutions were prepared fresh and used no longer than 5 hours before encapsulation in the HL-GUV.

Assembly of hydrogel-loaded giant unilamellar vesicles (HL-GUVs)

HL-GUVs were prepared by adapting our previously reported protocol for preparation of GUVs by the inverted emulsion method.[3] All lipids were prepared in stock solutions in chloroform, from which they were added to the paraffin oil. The main lipid components DOPC/POPC/Chol were combined in a 35/35/30 molar ratio in 200 μL of paraffin (total lipid concentration 10 mM). In addition, 1% of DSPE-PEG and 0.06% DOPE-RhB were added for membrane functionalization. This mixture was heated to 80 °C for 30 min in a sand bath. Inner phase solutions (20 μL) for HL-GUVs were prepared containing 200 mM sucrose and 10 μL of hydrogel precursor solution (final hydrogelator concentration in the inner phase=10 wt%). When required, urease (3.5 mg/mL), 0.1 mM pyranine, esterase (3.5 mg/mL), and/or HRP (3.5 mg/mL) were added to the inner phase. 20 μL of the inner phase were added to the paraffin oil suspension and vortexed for 25 seconds while turning the tube to prevent sedimentation. For enzyme activity experiments, PBS 1X was added in the inner and outer phases. The entire suspension was taken and layered on top of an outer phase solution in a tube (200 mM glucose, pH ~9). Subsequently, the tube was centrifuged at 3,300 g for 20 minutes at room temperature. The HL-GUVs were obtained by puncturing the tube at the bottom and obtaining the aqueous layer. The HL-GUVs were purified by centrifuging for 2 minutes at 1,500 g and carefully washing with outer phase, removing the supernatant. This washing step was performed twice. Preparation of giant unilamellar vesicles (GUVs) containing no hydrogel were performed in a similar manner, with the hydrogel precursor volume being replaced by basic MilliQ water.

Fluorescence recovery after photobleaching (FRAP) experiments

Using the confocal microscope, a circular area of 3 μm at the center of the selected vesicle was photo-bleached at 100% laser power (excitation at 638 nm) for 10 frames (1 frame/s). Subsequently, post-bleaching images were acquired at 0.5% laser power (3-10 min). Images were processed using the Leica Las X software. The fluorescence intensity was normalized by the prebleach steady state fluorescence intensity, correcting for

background fluorescence. The half time recovery and mobile fraction were determined by using the FrapBot software using a single exponential fitting.[4] For experiments depicted in Fig. 4, the pH of the environment was switched between basic (pH≥8.5) and acidic (pH≤7) by addition of small aliquots of HCl (0.1M) and NaOH (0.1M). For experiments depicted in Fig. 5, the pH of the environment was acidified by addition of small aliquots of HCl (0.1M); urea was added at a final concentration of 25 mM followed by incubation for at least 1 hour. FRAP measurements were performed at least three times using different vesicles. The intensity of the ratiometric pH probe HPTS (co-encapsulated in the HL-GUV lumen) was examined upon excitation at 405 nm and 488 nm.

Reaction kinetics experiments

For reaction kinetics experiments, inner and outer phases were supplemented with PBS (1x). After preparation, the prepared HL-GUVs were placed in PBS solution (1x, 200 mM glucose) at the corresponding pH (7 or 9). For calcein-AM conversion experiments, calcein-AM (400 nM) was added to the outer phase in a microscope well, followed by addition of HL-GUVs and incubation for 30 min. Calcein intensity (excitation at 488 nm, emission at 500-535 nm) was then monitored every 5 minutes by taking images at several spots containing HL-GUVs. For Amplex Red conversion experiments, HL-GUVs were placed in PBS solution (1x, 200 mM glucose) at pH 7 for 10 min followed by addition of urea (25 mM final concentration). After 1 hour incubation, Amplex Red (10 μM) and H_2O_2 (0.001%) were added and the resorufin intensity (absorbance at 572 nm) was monitored using a plate reader spectrophotometer (with spectra being taken every 3 minutes).

Table SI-1. Summary of reported artificial cell systems with regulation of catalytic function.

**Publication**	**Compartment membrane**	**Compartment core**	**Adaptation Mechanism**
This work	Lipidic	Responsive hydrogel	Cytosolic Viscoadaptation
H. Che *et al.*, *J. Am. Chem. Soc., 2018,* ***140****, 5356–5359.*	Polymeric	Aqueous solution	Membrane modulation
S. Cao *et al.*, *Angew. Chem. Int. Ed.*, 2022, **61**, e202205266.	Polymeric	Aqueous solution	Membrane modulation
M. J. Booth *et al.*, *Sci. Adv.*, 2016, **2**, 1–12.	Lipidic	Aqueous solution	Membrane modulation
S. Sun *et al.*, *Small*, 2016, **12**, 1920–1927.	Polymeric	Aqueous solution	Membrane modulation
C. Love *et al.*, *Angew. Chem. Int. Ed.*, 2020, **59**, 5950–5957.	Lipidic	Aqueous solution/coacervate droplets	Coacervation
W. Liu *et al.*, *J. Am. Chem. Soc.*, 2023, **145**, 22.	DNA	Aqueous solution/coacervate droplets	Coacervation

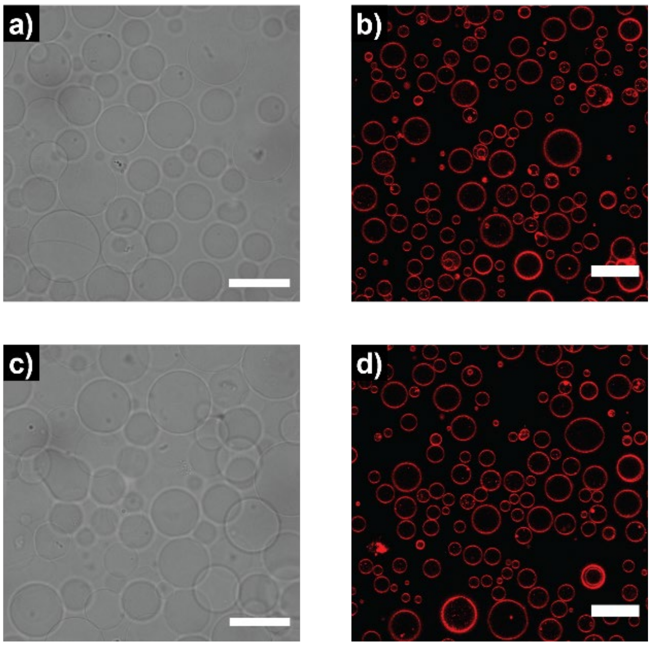

Fig. S1. (a) Bright field and (b) confocal microscopy images of GUVs prepared at pH 9. (c) Bright field and (d) confocal microscopy images of GUVs prepared at pH 7. Scale bars represent 30 μm.

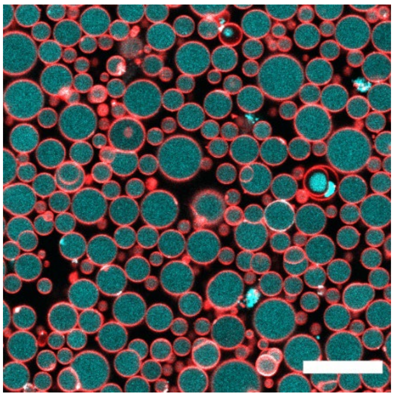

Fig. S2. Confocal image of HL-GUVs after reducing the pH to 7, shown as the overlay of the membrane and hydrogel markers (red: RhB-DOPE as marker of the lipid membrane, cyan: UPy-Cy5 as marker of the hydrogel phase). Scale bar represents 30 μm.

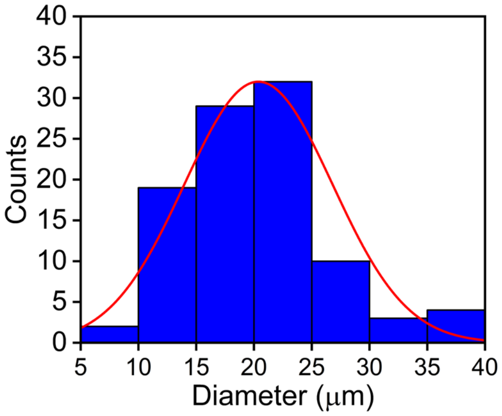

Fig. S3. Size distribution of HL-GUVs at pH 7, as determined from confocal microscopy analysis of multiple vesicles (N=100) using the software Fiji.

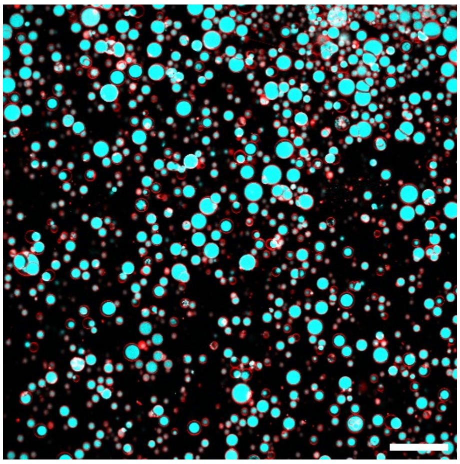

Fig. S4. Confocal image of HL-GUVs after reducing the pH to 3 (acquired using a 20x objective), shown as the overlay of the membrane and hydrogel markers (red: RhB-DOPE as marker of the lipid membrane, cyan: UPy-Cy5 as marker of the hydrogel phase). Scale bar represents 100 μm.

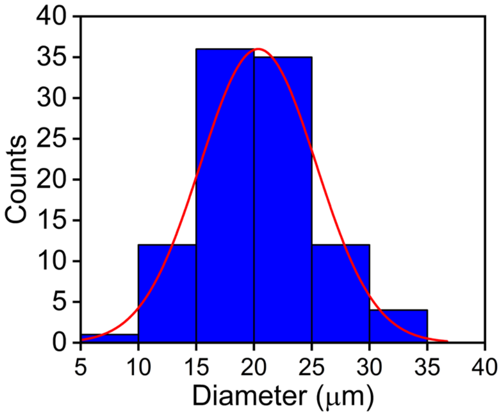

Fig. S5. Size distribution of HL-GUVs at pH 3, as determined from confocal microscopy analysis of multiple vesicles (N=100) using the software Fiji.

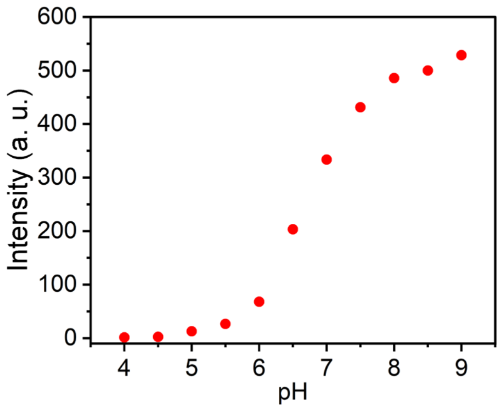

Fig. S6. HPTS fluorescence as a function of pH upon excitation at 480 nm (emission at 520 nm).

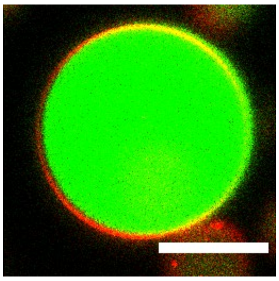

Fig. S7. Confocal micrograph of a HL-GUV showing HPTS fluorescence in green (excitation at 488 nm) under basic conditions (pH 9).

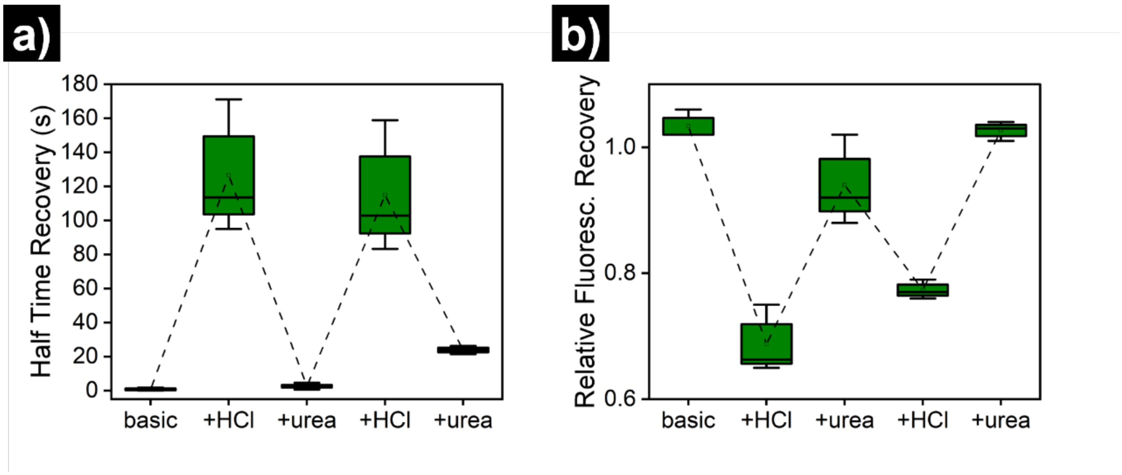

Fig. S8. (a) Half time recovery through multiple cycles upon subsequent addition of acid and urea, and (b) relative fluorescence recovery in the bleached area (10 min post-

bleaching). Data shown as box-plots from N≥3. Urease and HPTS (fluorescent pH probe) were co-encapsulated in the HL-GUV lumen.

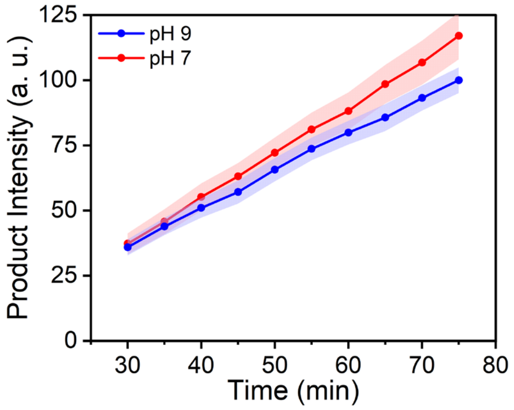

Fig. S9. Kinetics of product conversion by GUVs (without hydrogel) containing esterase (as catalyst that converts calcein-AM into fluorescent calcein) at pH 7 and pH 9. Data is represented as mean ± s. e., N≥8.

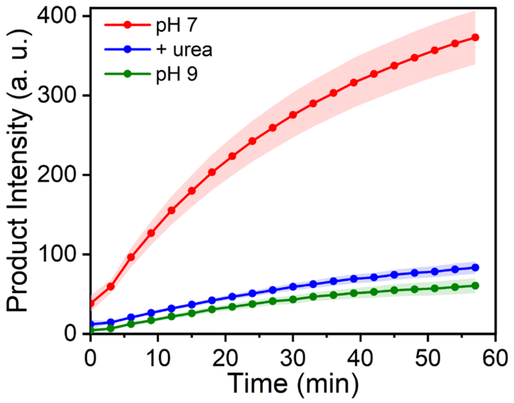

Fig. S10. Kinetics of product conversion by GUVs (without hydrogel) containing urease and peroxidase (as catalyst that converts Amplex Red into fluorescent resorufin) when set at pH 7, pH 7 upon addition of urea, and pH 9. Data is represented as mean ± s. e., N=3.

Supplementary References

1 I. de Feijter, O. J. G. M. Goor, S. I. S. Hendrikse, M. Comellas-Aragonès, S. H. M. Söntjens, S. Zaccaria, P. P. K. H. Fransen, J. W. Peeters, L. G. Milroy and P. Y. W. Dankers, *Synlett*, 2015, **26**, 2707–2713.

2 M. J. G. Schotman, M. M. C. Peters, G. C. Krijger, I. van Adrichem, R. de Roos, J. L. M. Bemelmans, M. J. Pouderoijen, M. G. T. A. Rutten, K. Neef, S. A. J. Chamuleau and P. Y. W. Dankers, *Adv Healthc Mater*, 2021, **10**, 2001987.

3 B. C. Buddingh', A. Llopis-Lorente, L. K. E. A. Abdelmohsen and J. C. M. van Hest, *Chem Sci*, 2021, **11**, 12829–12834.

4 R. Kohze, C. E. J. Dieteren, W. J. H. Koopman, R. Brock and S. Schmidt, *Cytometry A*, 2017, **91**, 810–814.
